# Mass Cytometry Analysis of the NK Cell Receptor–Ligand Repertoire Reveals Unique Differences between Dengue-Infected Children and Adults

**DOI:** 10.4049/immunohorizons.2000074

**Published:** 2020-10-16

**Authors:** Julia L. McKechnie, Davis Beltrán, Anne-Maud M. Ferreira, Rosemary Vergara, Lisseth Saenz, Ofelina Vergara, Dora Estripeaut, Ana B. Araúz, Laura J. Simpson, Susan Holmes, Sandra López-Vergès, Catherine A. Blish

**Affiliations:** * Program in Immunology, Stanford University School of Medicine, Stanford, CA 94305;; † Department of Research in Virology and Biotechnology, Gorgas Memorial Institute for Health Studies, Panama City, Panama;; ‡ Institute for Scientific Research and Technology Services, Panama City, Panama;; § Department of Biotechnology, Acharya Nagarjuna University, Guntur 522002, India;; ¶ Department of Statistics, Stanford University, Stanford, CA 94305;; ‖ Department of Medicine, Stanford University School of Medicine, Stanford, CA 94305;; # Hospital del Niño Doctor José Renán Esquivel, Panama City, Panama;; ** Hospital Santo Tomas, Panama City, Panama;; †† Universidad de Panama, Panama City, Panama

## Abstract

Dengue virus (DENV) is a significant cause of morbidity in many regions of the world, with children at the greatest risk of developing severe dengue. NK cells, characterized by their ability to rapidly recognize and kill virally infected cells, are activated during acute DENV infection. However, their role in viral clearance versus pathogenesis has not been fully elucidated. Our goal was to profile the NK cell receptor–ligand repertoire to provide further insight into the function of NK cells during pediatric and adult DENV infection. We used mass cytometry to phenotype isolate NK cells and PBMCs from a cohort of DENV-infected children and adults. Using unsupervised clustering, we found that pediatric DENV infection leads to a decrease in total NK cell frequency with a reduction in the percentage of CD56^dim^CD38^bright^ NK cells and an increase in the percentage of CD56^dim^perforin^bright^ NK cells. No such changes were observed in adults. Next, we identified markers predictive of DENV infection using a differential state test. In adults, NK cell expression of activation markers, including CD69, perforin, and Fas-L, and myeloid cell expression of activating NK cell ligands, namely Fas, were predictive of infection. In contrast, increased NK cell expression of the maturation marker CD57 and myeloid cell expression of inhibitory ligands, such as HLA class I molecules, were predictive of pediatric DENV infection. These findings suggest that acute pediatric DENV infection may result in diminished NK cell activation, which could contribute to enhanced pathogenesis and disease severity.

## INTRODUCTION

Dengue virus (DENV), a flavivirus with four serotypes (DENV1–4), is the most prevalent arthropod-borne virus in the world. Infection begins when an individual is bitten by a DENV-infected *Aedes* mosquito. After an incubation period of 4–10 d, a majority of DENV-infected individuals will develop an asymptomatic infection or mild symptoms associated with dengue fever such as fever, headache, vomiting, myalgia, and rash. Generally, these symptoms persist for 3–7 d before patients enter into defervescence. However, upon defervescence, a small percentage of patients develop severe dengue characterized by severe plasma leakage, hemorrhage, and/or organ impairment (1).

DENV infection presents differently in children and adults. Vomiting, skin rash, abdominal pain, and anorexia are commonly observed in children, whereas myalgia, nausea, retro-orbital pain, arthralgia, headache, and leukopenia are symptoms typical of adult DENV infection (2–5). Interestingly, children under the age of 16 are not only more likely to develop symptomatic dengue; they are also more likely to develop severe dengue and succumb to the infection (2, 6–10). There are several potential reasons as to why this is the case. The increase in plasma leakage observed in DENV-infected infants and children could be explained by higher capillary fragility (11). Additionally, Ab-dependent enhancement caused by waning maternal Abs or secondary DENV infection may contribute to increased disease severity (2, 12–14). Although increased capillary fragility and Ab-dependent enhancement could both be contributing factors, increased risk of severe dengue in children compared with adults may also be due to differences in the immune response.

The evolution of the immune system with aging, as well as its implications for antiviral immunity, has been well studied (15, 16). Broadly, people are born with an immature Immune system that, with age, matures and develops immunological memory to previously encountered viruses. Traditionally, immune experience is strictly thought to shape the B and T cell repertoire. However, a previous study has demonstrated that immune experience acquired throughout life results in an increase in the diversity of NK cells (17), an innate immune cell subset that acts as one of the first responders to viral infection. Furthermore, numerous studies in the past decade have also revealed the ability of NK cells to develop both Ag-dependent and Ag-independent immunological memory (18).

NK cells kill infected target cells via three mechanisms: degranulation with release of cytotoxic mediators, receptor-mediated apoptosis, and Ab-dependent cellular cytotoxicity. NK cells are activated to kill or secrete cytokines based on activating and inhibitory signals received from germline-encoded receptors binding to their cognate ligands on potential target cells. Although NK cells are known to be activated during DENV infection, particularly during the acute phase (19–23), it is unclear which NK cell subsets are actually responding. Some putative receptor-ligand interactions that may trigger an anti-DENV NK cell response such as NKp44/E protein, KIR2DS2/NS3-HLA-C, and others have been reported (24–26). We and others have also shown that upregulation of HLA class I molecules by DENV-infected cells suppresses the NK cell response (27–29). Importantly, prior work investigating the role of NK cells during in vivo DENV infection has been limited to examining either pediatric or adult patients, but never both in parallel (20–23, 30).

Our goal was to determine whether NK cells in children and adults respond differently to acute DENV infection. Using a cohort of pediatric and adult DENV patients from Panama, a dengue-endemic country, we profiled the expression of NK cell receptors and their ligands by mass cytometry (CyTOF). We found that acute DENV infection in children leads to a decrease in NK cell frequency, shifts in the composition of the NK cell compartment, as well as NK cell maturation marked by increased CD57 expression. No changes in NK cell frequency occurred in adults. However, DENV infection did result in increased expression of NK cell activation and functional markers, CD69, perforin, and Fas-L. Finally, analysis of myeloid cell subsets identified by unsupervised clustering revealed that DENV infection leads to a more dramatic departure from baseline phenotype in children, with greater induction of ligands for inhibitory NK cell receptors than observed in adults. Overall, this work suggests that pediatric DENV infection may result in a more suppressed NK cell response compared with adult DENV infection.

## MATERIALS AND METHODS

### DENV cohort and ethical statement

Adult and pediatric patients experiencing symptoms of acute DENV infection for 5 d or less were enrolled at public health institutions in Panama City and surrounding suburban areas in Panama from 2013 to 2015. Healthy control samples were collected from volunteers at Gorgas Memorial Institute of Health Studies (ICGES). Patients were considered positive for dengue infection if they had a positive RT-PCR result. Suspected DENV patients negative for DENV, Chikungunya virus, and Zika virus were considered undifferentiated febrile patients. All dengue patients were infected with DENV-2, with the exception of one adult patient who was infected with DENV-1. Pediatric and adult DENV cases were mild and largely the result of primary infections. None of the patients progressed to severe disease. The Institutional Review Board of Hospital del Niño (CBIHN-M-0634) approved this study protocol. Committees of ICGES, Caja de Seguro Social, Santo Tomas Hospital, and Stanford University confirmed the protocol.

### PBMC sample processing, storage, and thawing

PBMCs were collected using Ficoll-Paque. Following isolation, PBMCs were stored at −80°C for 24–72 h in freezing media (90% FBS, 10% DMSO) before being transferred to liquid nitrogen. Prior to use, PBMCs were thawed in complete media (RPMI-1640, 10% FBS, 1% l-glutamine, 1% penicillin/streptomycin), centrifuged, and counted using a TC20 automated cell counter (Bio-Rad Diagnostics). One million PBMCs from each donor were set aside and kept on ice for ligand staining. NK cells were isolated from the remaining PBMCs by negative selection using a human NK Cell Isolation Kit (Miltenyi). Following NK cell isolation, NK cells were centrifuged and counted.

### CyTOF staining, data acquisition, and processing

Cells were stained for CyTOF as previously described (29). Briefly, in-house conjugated Abs were made using Maxpar X8 Ab Labeling Kits (Fluidigm), whereas preconjugated Abs were purchased from Fluidigm and Thermo Fisher Scientific. PBMCs and isolated NK cells were stained using the viability marker cisplatin (Enzo Life Sciences). They were then CD45 barcoded with a two-of-four palladium barcoding scheme using ^102^Pd, ^104^Pd, ^106^Pd, and ^108^Pd. NK cells were not isolated from donors with fewer than 2 million viable PBMCs. Barcoded samples were pooled and stained with surface Abs before fixation with 2% paraformaldehyde and permeabilization (eBioscience Permeabilization Buffer). Samples were then stained with intracellular Abs and incubated in iridium-191/193 intercalator (DVS Sciences) for up to a week. Before the analysis by CyTOF, samples were washed and diluted in EQ Four Element Calibration Beads. FCS files were normalized, and calibration beads were removed using the ParkerICI Premessa package. Normalized data were then debarcoded using the same Premessa package. FlowJo 10.2 was used to gate on cell subsets of interest. Samples with <50% viability by TC20 automated cell counter were excluded from subsequent analysis.

### CyTOF data analysis

The open source statistical software R (https://www.r-project.org/, version 3.6.1) (31) was used to perform the CyTOF data analysis. To account for heteroskedasticity, the signal intensities were transformed using the hyperbolic sine transformation (cofactor equals to 5) prior to any downstream analysis.

#### Clustering.

**FIGURE 1.**
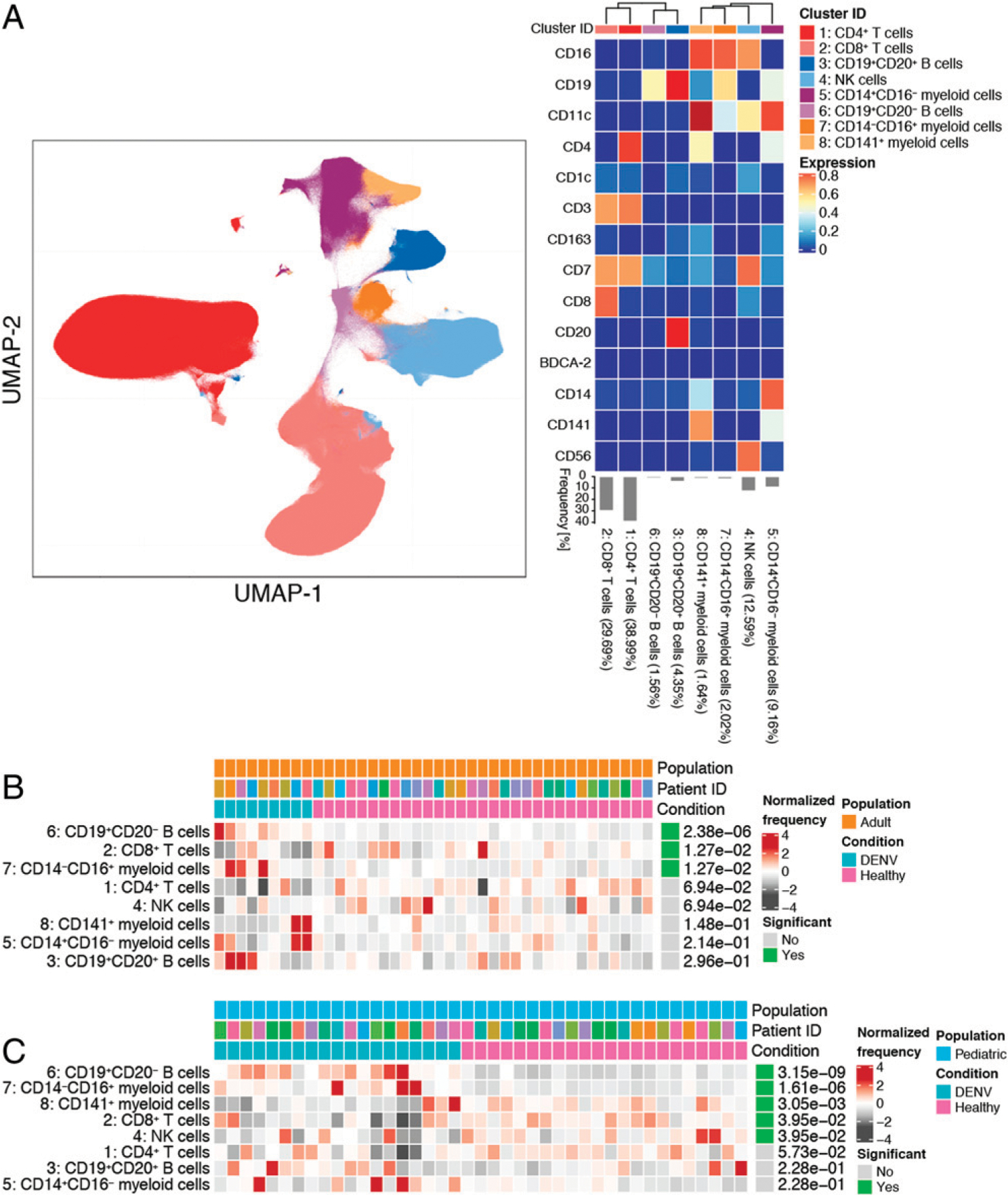
Acute DENV infection alters the frequencies of immune cell subsets. (**A**) UMAP visualization of PBMCs from DENV^+^, undifferentiated febrile illness, and healthy control pediatric and adult groups. The identity of each subset was determined based on expression of 14 lineage markers shown in the heat map. Each immune cell subset is color coded according to the key on the right. The heat map shows the expression of each marker (value scaled from 0 to 1) in each cluster. Clusters were hierarchically ordered based on similarity (dendrogram calculated using Euclidean distance as a metric and average as a linkage). The percentages associated with each cluster are the average of all pediatric and adult groups. (**B**) Results from differential abundance tests comparing the frequencies of immune cell subsets in DENV-infected adults (teal, *n* = 9) to healthy adults (pink, *n* = 31). (**C**) Results from a differential abundance test comparing the frequencies of immune cell subsets in DENV-infected children (teal, *n* = 19) to healthy children (pink, *n* = 22). Subsets whose frequencies were significantly different (adjusted *p* value < 0.05) between the two states are denoted by green boxes. The proportions of each cluster in each sample are represented by the normalized frequencies. Gray boxes correspond to under representation and red boxes correspond to over representation. The frequencies were scaled using arcsine-square-root transformation and then z-score normalized in each cluster (B and C).

The package *CATALYST* (version 1.10.1) (32) was used to perform the unsupervised clustering. We used 14 lineage markers (refer to Fig. 1A for details) for the PBMCs and 35 markers (refer to Fig. 3A for details) for the isolated NK cells. The default parameters of the clustering function were used. Briefly, this clustering method combines two algorithms: *FlowSOM* (33), which clusters the data into 100 high-resolution clusters, and *ConsensusClusterPlus* (34), which groups these high-resolution clusters into metaclusters. To determine the optimal number of metaclusters, we used the δ-area plot provided by the package at the end of the clustering step (eight metaclusters for the PBMCs; seven metaclusters for the isolated NK cells).

#### Uniform Manifold Approximation and Projection visualizations.

We used the *uwot* package (version 0.1.5 available on CRAN) (35), which implements the Uniform Manifold Approximation and Projection (UMAP) algorithm (L. McInnes, J. Healy, and J. Melville, manuscript posted on arXiv, DOI: arXiv: 1802.03426v3). Using the same markers previously used for the clustering, we applied this dimensionality reduction method with the following parameters: 0.1 as the minimum distance and 20 as the number of nearest neighbors.

#### Differential abundance tests.

Differential abundance tests were performed using the *diffcyt* package (version 1.6.1) (36) to identify differences in the frequencies of the cell clusters. We used the *diffcyt-DA-voom* function with the default parameters. Briefly, this method transforms the cluster cell counts to stabilize the mean/variance relationship with the *voom* method and then fits one linear model for each cluster. Each differential abundance test was performed on data that were filtered to the comparison of interest (adult or pediatric population; healthy or DENV patients). The corresponding design matrix and contrast matrix were generated for each comparison. The reported *p* values were adjusted by the false discovery rate approach.

#### Differential state tests.

To identify which markers were predictive of a specific state (e.g., healthy or DENV), we used the *CytoGLMM* package available on Github (Ref. 37 and C. Seiler, L.M. Kronstad, L.J. Simpson, M. Le Gars, E. Vendrame, C.A. Blish, and S. Holmes, manuscript posted on arXiv, DOI: arXiv:1903.07976v1). The method for unpaired samples implemented in this package uses a generalized linear model with bootstrap resampling to estimate the donor effect. The results of the model are the log-odds that a given marker is predictive of a specific state with a 95% confidence interval. The *p* values are computed using Efron and Tibshirani (38) methodology and corrected for multiple testing by Benjamini-Hochberg method. Thirty-five markers were considered for the differential state tests within the isolated NK cells (refer to Fig. 4 for details). Twenty-seven markers were considered for the differential state tests within the myeloid cell clusters of the PBMCs (refer to Fig. 5 for details). The following parameters were used: 2000 bootstraps, no subsampling was performed, and all samples were used (i.e., no threshold on the minimum number of cells per sample).

## RESULTS

### Effects of DENV infection on immune cell subsets

Given the differences in disease course between DENV-infected children and adults, as well as the impacts of age on the antiviral immune response, we evaluated whether there were shifts in the abundances of immune cell subsets in Panamanian adult and pediatric DENV patients during the acute phase of infection. PBMCs were isolated from nine DENV^+^ adults, 31 healthy adults, 19 DENV^+^ children, and 22 healthy children (Table I). All DENV cases were mild, and with the exception of one adult DENV-1 patient, were caused by DENV-2 infection. A majority of the cases were the result of a primary infection. PBMC samples were stained for CyTOF (Supplemental Table I) and gated down to live cells (Supplemental Fig. 1). Unsupervised clustering of live cells identified eight canonical immune cell subsets: CD4^+^ T cells (cluster 1, 38.99%), CD8^+^ T cells (cluster 2, 29.69%), CD19^+^CD20^+^ B cells (cluster 3, 4.35%), NK cells (cluster 4, 12.59%), CD14^+^CD16^−^ myeloid cells (cluster 5, 9.16%), CD19^+^CD20^−^ B cells (cluster 6, 1. 56%), CD14^−^CD16^+^ myeloid cells (cluster 7, 2.02%), and CD141^+^ myeloid cells (cluster 8, 1.64%) (Fig. 1A). A differential abundance test revealed that DENV infection in both children and adults leads to a significant increase in the frequencies of CD19^+^CD20^−^ B cells and CD14^−^CD16^+^ myeloid cells as well as a decrease in CD8^+^ T cell frequency compared with healthy controls (Fig. 1B, 1C). Unlike adults, children also demonstrated decreases in the frequencies of CD141^+^ myeloid cells and NK cells, although there was heterogeneity between individuals (Fig. 1C). These data indicate that, in general, acute DENV infection has a broader impact on immune cell subsets in children than adults.

**FIGURE 2.**
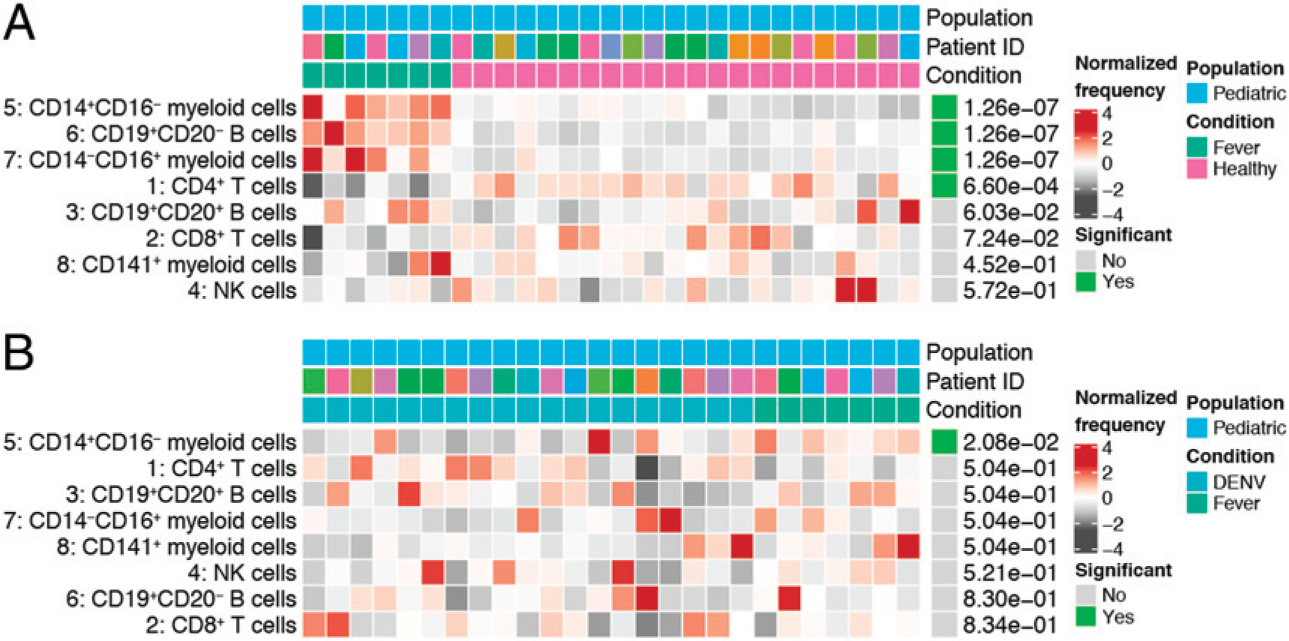
Undifferentiated febrile illness in pediatric patients uniquely affects the frequencies of specific immune cell subsets. (**A**) Results from a differential abundance test comparing the frequencies of immune cell subsets in children presenting with an undifferentiated febrile illness (green, *n* = 7) to healthy children (pink, *n* = 22). (**B**) Results from a differential abundance test comparing the frequencies of immune cell subsets in DENV-infected children (teal, *n* = 19) and children presenting with an undifferentiated febrile illness (green, *n* = 7). Subsets whose frequencies were significantly different (adjusted *p* value < 0.05) between the two states are denoted by green boxes. The proportions of each cluster in each sample are represented by the normalized frequencies. Gray boxes correspond to underrepresentation and red boxes correspond to overrepresentation. The frequencies were scaled using arcsine-square-root transformation and then z-score normalized in each cluster (A and B).

Our cohort also included seven children presenting with an undifferentiated febrile illness who were determined to be DENV^−^ by RT-PCR. To identify features associated with febrile illness, we performed a differential abundance test comparing these undifferentiated febrile patients to healthy children (Fig. 2A). Similar to our DENV^+^ versus healthy children comparison, we observed significant increases in the frequencies of CD19^+^CD20^−^ B cells and CD14^−^CD16^+^ myeloid cells in undifferentiated febrile patients compared with healthy controls. An increase in CD14^+^CD16^−^ myeloid cell frequency and a decrease in CD4^+^ T cell frequency were also observed. Notably, unlike DENV infection, undifferentiated febrile illness had no impact on the frequency of NK cells when compared with healthy controls. To identify features associated explicitly with DENV infection, we performed a differential abundance test comparing DENV-infected children to undifferentiated febrile children (Fig. 2B). This revealed that the frequency of CD14^+^CD16^−^ myeloid cells was significantly lower in children infected with DENV. Therefore, whereas both DENV infection and undifferentiated febrile illness uniquely impact certain immune cell subsets compared with healthy children, only an increase in CD14^+^CD16^−^ myeloid cell frequency differentiates other febrile illnesses from DENV infection.

Given the importance of NK cells during the acute phase of viral infection, we were intrigued by the decrease in NK cell frequency observed in pediatric DENV cases. Consequently, we performed a deeper analysis of the NK cell compartment.

### Identification of NK cell subsets and the impact of DENV infection on their frequencies

**FIGURE 3.**
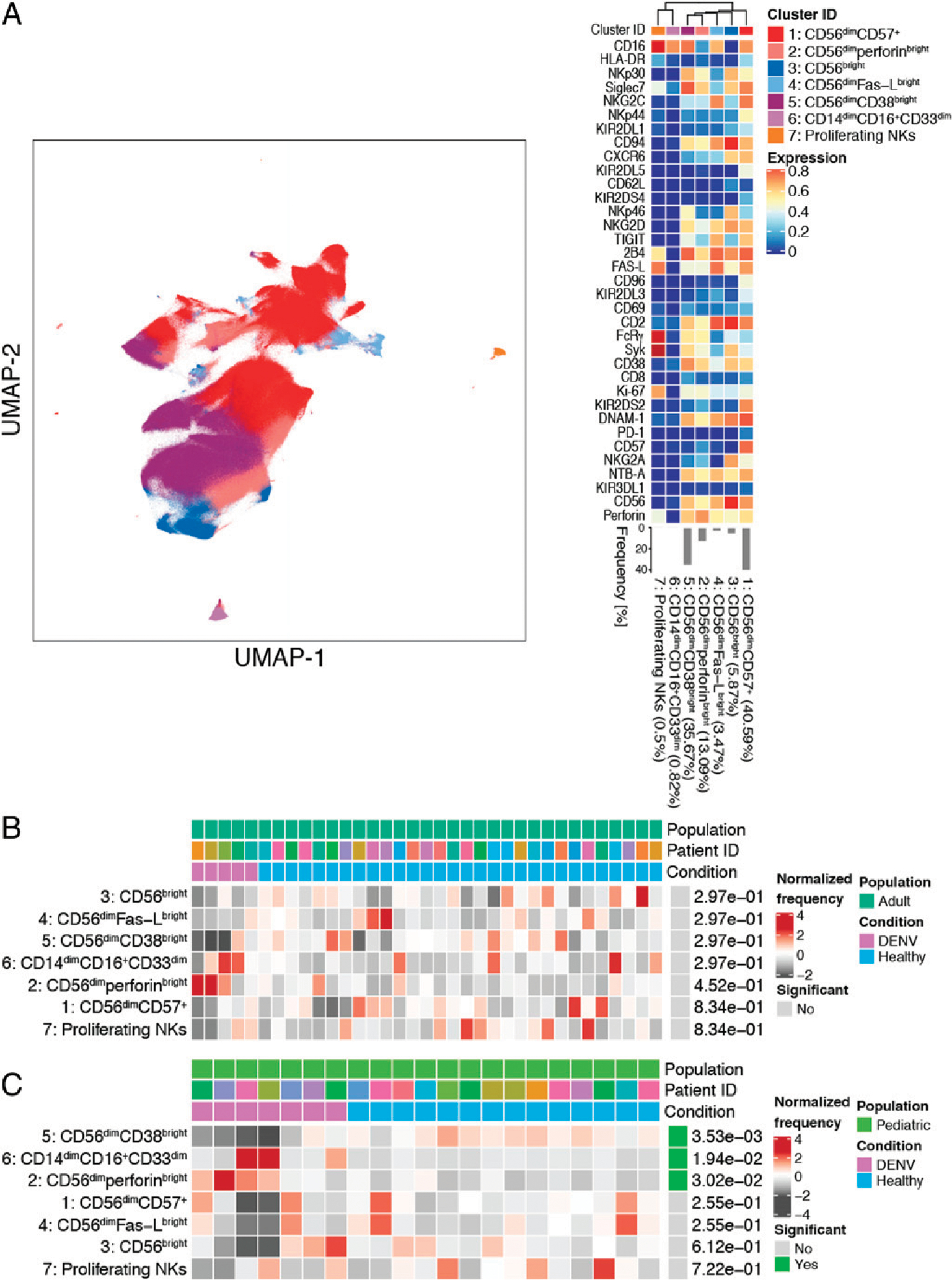
Acute DENV infection changes the composition of the NK cell compartment in children but not adults. (**A**) UMAP visualization of NK cells from DENV^+^ and healthy control pediatric and adult groups. The identity of each NK cell subset was determined based on the expression of 35 NK cell markers shown in the heat map. Each immune cell subset is color coded according to the key to the right of the heat map. The heat map shows the expression of each marker (value scaled from 0 to 1) in each cluster. Clusters were hierarchically ordered based on similarity (dendrogram calculated using Euclidean distance as a metric and average as a linkage). The percentages associated with each cluster are the average of all pediatric and adult groups. (**B**) Results from a differential abundance test comparing the frequencies of NK cell subsets in DENV-infected adults (purple, *n* = 5) to healthy adults (blue, *n* = 30). (**C**) Results from a differential abundance test comparing the frequencies of immune cell subsets in DENV-infected children (purple, *n* = 7) to healthy children (blue, *n* = 14). NK cell subsets whose frequencies were significantly different (adjusted *p* value < 0.05) between the two states are denoted by green boxes. The proportions of each cluster in each sample are represented by the normalized frequencies. Gray boxes correspond to underrepresentation and red boxes correspond to overrepresentation. The frequencies were scaled using arcsine-square-root transformation and then z-score normalized in each cluster (B and C).

To identify NK cell subsets responding to DENV infection, we performed unsupervised clustering of purified NK cells. NK cells were isolated from donors with at least 2 million viable PBMCs and stained with an NK cell–focused CyTOF panel (Supplemental Table II). Negative gating was performed before downstream analysis to remove any residual, non–NK cells (Supplemental Fig.2). Our analysis identified the canonical CD56^bright^ and CD56^dim^ NK cell subsets (Fig. 3A). Although CD56^bright^ NK cells clustered together (cluster 3, 5.87%), CD56^dim^ NK cells were divided among four clusters. The largest CD56^dim^ clusters (clusters 1 and 5) consisted of CD56^dim^CD57^+^ (40.59%) and CD56^dim^CD38^bright^ (35.67%) NK cells, respectively. The less abundant CD56^dim^ subsets corresponding to clusters 2 (13.09%) and 4 (3.47%) consisted of CD56^dim^perforin^bright^ and CD56^dim^Fas-L^bright^ NK cells, respectively. Two clusters, clusters 6 and 7, each made up <1% of the total NK cells. Of the 35 NK cell markers examined, cluster 6 only expressed one (CD16), making it likely that this cluster represents CD14^dim^CD16^+^CD33^dim^ monocytes that were not removed by purification or negative gating (Supplemental Fig. 2). Cells in cluster 7 express the NK cell markers 2B4, Fas-L, FcRγ, Syk, Ki-67, and CD16. Therefore, it is likely that this cluster represents a small population of actively proliferating NK cells.

Next, we performed a differential abundance test to determine whether the frequencies of the identified NK cell subsets change during acute DENV infection. Interestingly, there were no significant differences in the composition of the NK cell compartment between DENV-infected adults and healthy adult controls (Fig. 3B). However, in children, DENV infection resulted in a significant decrease in the frequency of CD56^dim^CD38^bright^ NK cells and an increase in the frequency of CD56^dim^perforin^bright^ NK cells (Fig. 3C). There was also an increase in the abundance of the second smallest cluster (cluster 6), which, as previously discussed, is likely made up of CD14^dim^CD16^+^CD33^dim^ monocytes.

### Effects of DENV infection on NK cell phenotype

**FIGURE 4.**
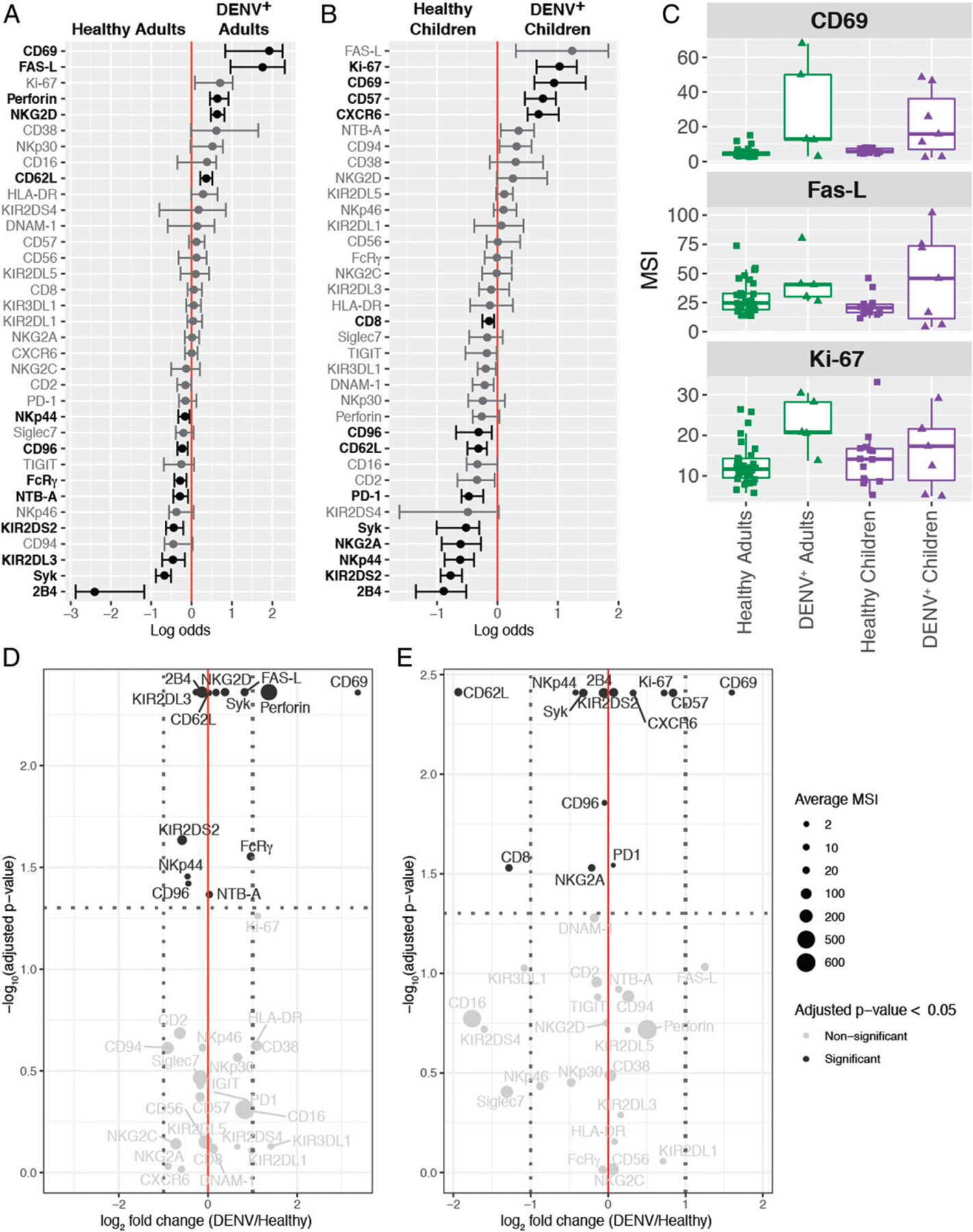
Acute DENV infection results in an activated NK cell phenotype in adults and NK cell maturation in children. (**A**) A generalized linear model with bootstrap resampling was used to identify markers on total NK cells that were predictive of adult DENV infection (right, *n* = 5) and healthy adults (left, *n* = 30). Black bars represent the 95% confidence interval. (**B**) A generalized linear model with bootstrap resampling was used to identify markers on total NK cells that were predictive of pediatric DENV infection (right, *n* = 7) and healthy children (left, *n* = 14). Markers with an adjusted *p* value <0.05 are shown in black and markers with an adjusted *p* values >0.05 are shown in gray (A and B). (**C**) Box plots showing the MSI for the top three markers associated with DENV infection in both adults and children. Healthy adults (*n* = 30) are shown in green squares, DENV^+^ adults (*n* = 5) are shown in green triangles, healthy children (*n* = 14) are shown in purple squares, and DENV^+^ children (*n* = 7) are shown in purple triangles. (**D**) Volcano plot for adult NK cells illustrating markers whose adjusted *p* values in (A) were <0.05 in black and markers whose adjusted *p* values were >0.05 in gray. The horizontal dashed line marks the 0.05 *p* value cutoff. The −log_10_ *p* value for each marker is shown on the *y*-axis. The DENV/healthy log_2_ fold change for each marker is shown on the *x*-axis. The two vertical dashed lines provide a reference point for markers whose expression is increased 2-fold. The size of each point corresponds to the MSI for that specific marker. (**E**) Same as in (D), but for pediatric NK cells. The MSIs are assigned according to the key at the right and they correspond to the raw (untransformed) MSI. The reported *p* values are the adjusted *p* values generated by the generalized linear model with bootstrap resampling (D and E).

After observing significant shifts in the pediatric NK cell compartment upon DENV infection, we performed a differential state test to compare the phenotype of total NK cells in DENV-infected adults and children to their respective healthy controls (Fig. 4A, 4B). Interestingly, three markers of NK cell activation, CD69, Fas-L, and Ki-67, were associated with acute dengue in both adult and pediatric patients. These results are consistent with what has been previously published (19, 20, 22, 23). However, the extent to which NK cells upregulate these proteins varies between adults and children (Fig. 4C). There is also some heterogeneity within each of the DENV-infected groups.

We then visualized our NK cell CyTOF data using volcano plots, allowing us to compare each marker’s mean signal intensity (MSI), fold change (DENV/healthy), and how predictive it was of either state (DENV^+^ or healthy). As expected, DENV-infected adults had a >10-fold increase in CD69 expression (Fig. 4D). Adult DENV infection was also characterized by robust expression of perforin, as well as a modest increase in expression of death ligand Fas-L, and activating receptor NKG2D. CD69, perforin, Fas-L, and NKG2D were all predictive of adult DENV infection. These data suggest that adult NK cells are activated upon DENV infection and likely capable of responding to DENV-infected cells.

Interestingly, DENV infection in children seemed to result in a mature NK cell phenotype (Fig. 4E). Expression of the maturation marker CD57 increased nearly 2-fold in DENV-infected children and was significantly predictive of infection, whereas expression of the immaturity marker NKG2A was reduced. CD69 was also upregulated in DENV-infected children, albeit to a lesser extent than in DENV-infected adults (2.8-fold versus 10-fold). Other proteins whose expression increased upon DENV infection in children were chemokine receptor CXCR6 and proliferation marker Ki-67. The expression of death receptor PD-1 was also slightly increased. CD57, CD69, CXCR6, Ki-67, and PD-1 were all significantly predictive of pediatric DENV infection. Together, these data suggest that DENV infection may drive maturation and modest activation of NK cells in children.

### Myeloid cell expression of ligands for NK cell receptors

**FIGURE 5.**
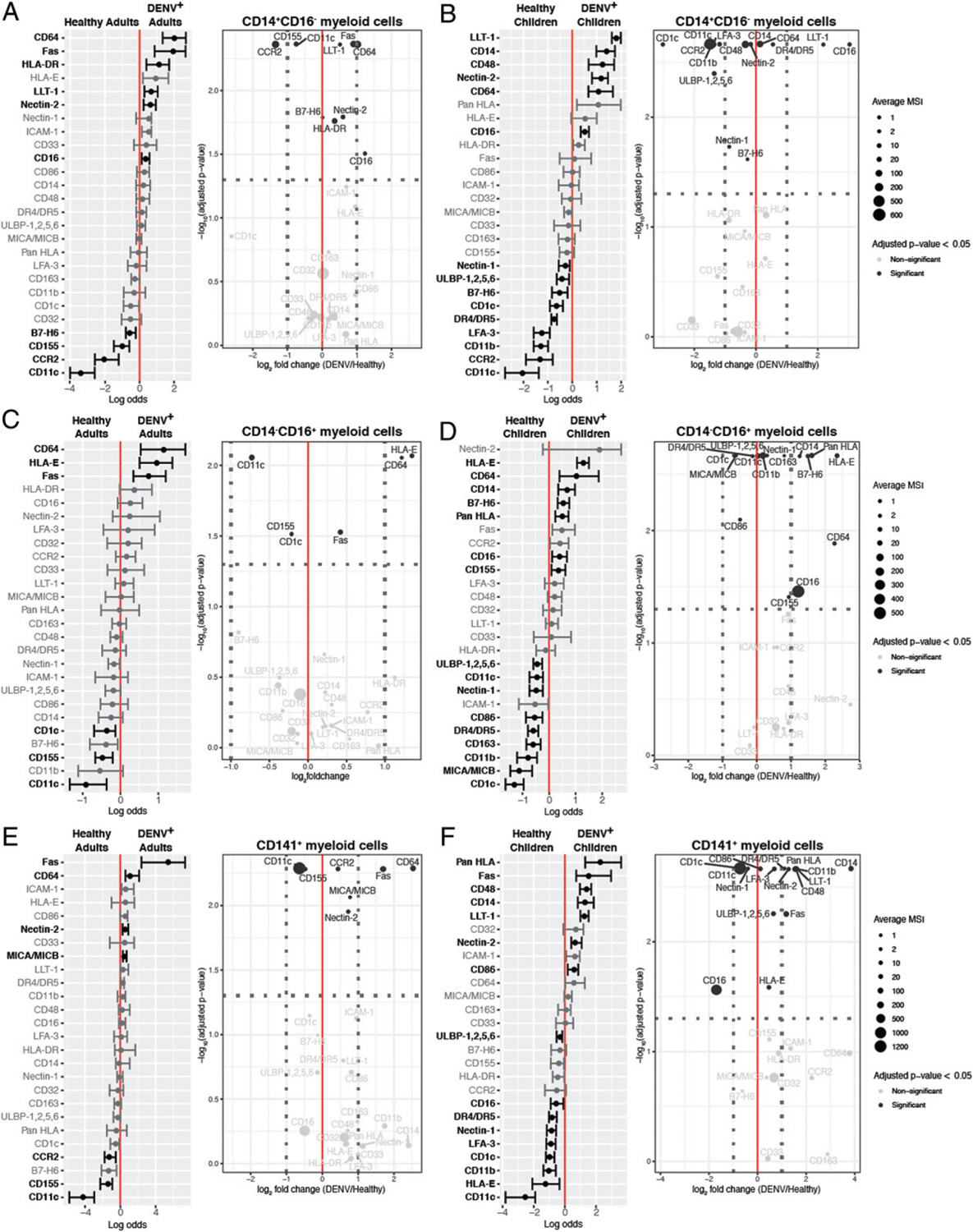
Pediatric DENV infection results in a dramatic shift in the phenotype of myeloid subsets and increased expression of inhibitory ligands compared with adult DENV infection. A generalized linear model with bootstrap resampling was used to identify markers on adult CD14^+^CD16^−^ (**A**), CD14^−^CD16^+^ (**C**), and CD141^+^ (**E**) myeloid cells and pediatric CD14^+^CD16^−^ (**B**), CD14^−^CD16^+^ (**D**), and CD141^+^ (**F**) myeloid cells that were predictive of DENV infection (right, *n* = 9 adults or 19 children) and healthy patients (left, *n* = 31 adults or 22 children). Volcano plots accompany each generalized linear model. The horizontal dashed line marks the 0.05 *p* value cutoff. The −log_10_ *p* value for each marker is shown on the *y*-axis. The DENV/healthy log_2_ fold change for each marker is shown on the *x*-axis. The two vertical dashed lines provide a reference point for markers whose expression is increased 2-fold. The size of each point corresponds to the MSI for that specific marker. The MSI key is the same for adult and pediatric myeloid cells within the same subset and they correspond to the raw (untransformed) MSI. Both the generalized linear models and volcano plots illustrate markers whose adjusted *p* values were <0.05 in black and markers whose adjusted *p* values were >0.05 in gray.

Given our finding that NK cells are activated to some extent during acute DENV infection irrespective of patient age, and our identification of a CD56^dim^perforin^high^ NK cell subset, we next sought to identify ligands for NK cell receptors that could contribute to their activation. As myeloid cells are the main targets of DENV infection (39), we focused on the three myeloid subsets identified by our unsupervised clustering: CD14^+^CD16^−^, CD14^−^CD16^+^, and CD141^+^ myeloid cells. Unfortunately, we were unable to detect DENV proteins and therefore could not compare ligand expression by infected and uninfected cells. In the adult CD14^+^CD16^−^ subset, there were 10 markers whose expression was significantly predictive of either DENV infection or healthy controls (Fig. 5A). The markers that were upregulated during DENV infection included death receptor Fas, inhibitory CD161 ligand LLT-1, and HLA-DR. In contrast, there were 15 markers whose expression was significantly altered in children (Fig. 5B). Markers that were upregulated during pediatric DENV infection included LLT-1 and death receptor 4/5 (DR4/DR5).

In the CD14^−^CD16^+^ subset, there were six markers whose expression was significantly altered in adults (Fig. 5C). The markers whose expression was upregulated during DENV infection included Fas as well as HLA-E, whose role in suppressing the NK cell response to DENV-infected cells is currently unclear (29). In the pediatric CD14^−^CD16^+^ myeloid cell subset, there were 16 significant markers (Fig. 5D). Those whose expression was higher during DENV infection included the well-known NK cell inhibitory ligands, HLA class I molecules, as well as known activating NKp30 and NKG2D ligands, B7-H6 and ULBP-1,2,5,6, respectively.

Finally, in the CD141^+^ myeloid subset, there were seven significant markers in adults (Fig. 5E). Those that were upregulated during DENV infection included Fas as well as MICA/MICB, ligands for the activating NK cell receptor NKG2D. There were 16 significant markers in children (Fig. 5F). Inhibitory ligands DR4/DR5, LLT-1, and HLA class I molecules as well as activating ligands Fas and ULBP-1, -2, -5, and -6, along with the costimulatory marker CD86, were among those upregulated during pediatric DENV infection. These findings suggest that pediatric DENV infection leads to more significant changes in myeloid cell phenotype compared with adult infection. The phenotype of adult myeloid cells likely promotes NK cell activation, whereas the phenotype of pediatric myeloid cells likely dampens NK cell activation.

Based on the work done by Costa et al. (26) demonstrating the importance of DNAM-1, 2B4, LFA-1, and CD2 in mediating the NK cell response to DENV-infected cells, we also looked at the impact of DENV infection on expression of their respective ligands. We found no consistent pattern in DNAM-1 ligands, Nectin-2, and CD155 (PVR), being associated with either the DENV^+^ or healthy state. The same was true for 2B4 and CD2 ligands, CD48 and LFA-3, respectively. Expression of ICAM-1, the LFA-1 ligand, was never significantly predictive of either state.

## DISCUSSION

Although severe dengue has historically been considered a children’s disease, there has been a recent increase in the average age of reported dengue cases, particularly in Southeast Asia (7, 40, 41). Considering that the geographical range of DENV is expected to expand in the coming years (42), increasing the portion of the population at risk for infection, it is important to determine whether there are age-related differences in how the immune system responds to DENV infection. Previous studies investigating the immune response to DENV infection have reported an increase in activated NK cells (19, 20, 22, 23, 30), suggesting a role for this immune cell subset. However, all of these studies solely focused on either pediatric or adult DENV infection, not both. In this study, we evaluated the NK cell receptor–ligand repertoire of pediatric and adult DENV patients by CyTOF. We show that, compared with adults, children have a lower frequency of NK cells and significant changes in the composition of their NK cell compartment. Whereas DENV infection leads to a markedly activated NK cell phenotype in adults, it results in NK cell maturation and modest activation in children. Additionally, acute pediatric DENV infection leads to significant myeloid cell upregulation of inhibitory NK cell ligands, which is largely absent in adult DENV patients. Together, these findings suggest that compared with adults, DENV infection in children favors a more suppressed NK cell response.

Increases in monocyte, activated T cell, NK cell, and plasmablast frequencies as well as decreases in the frequencies of total CD3^+^ and CD4^+^ T cells during acute DENV have been previously reported (30, 43, 44). We observed similar results in our cohort with DENV-infected children and adults experiencing a decrease in the frequency of CD8^+^ T cells and increases in the frequencies of CD19^+^CD20^−^ B cells and CD14^−^CD16^+^ myeloid cells. In addition, we observed a decrease in the frequencies of CD141^+^ myeloid cells and NK cells during acute pediatric DENV infection. These changes in the CD141^+^ myeloid cell and NK cell compartments were not observed when children presenting with an undifferentiated febrile illness were compared with healthy children. One subset that is notably absent from our clustering analysis is CD14^+^CD16^+^ myeloid cells. Studies have found that the frequency of CD14^+^CD16^+^ monocytes increases during DENV infection and may stimulate differentiation of plasmablasts (45,46). Our inability to detect this subset could be due to its low frequency compared with the other two monocyte subsets (47) or the fact that expansion of CD14^+^CD16^+^ monocytes typically occurs within the first 2 d of infection before dramatically dropping off (46).

Unsupervised clustering of isolated NK cells identified six NK cell subsets. Of the NK cell subsets identified, one expressed high levels of the functional marker perforin. In addition to perforin, the CD56^dim^perforin^bright^ subset expressed NKp30, Siglec-7, CD94, 2B4, CD2, FcRγ, CD38, Ki-67, DNAM-1, and NTB-A. CD38 and Ki-67 expression suggests this is an activated NK cell subset. This is further supported by the fact that these cells also express Siglec-7, which has been reported as a marker of highly functional NK cells (48). Moreover, in vitro studies have identified 2B4, CD2, and DNAM-1 as playing significant roles in mediating NK cell interactions with DENV-infected monocyte-derived dendritic cells (26). Blocking these proteins with Abs resulted in decreased NK cell expression of CD69 as well as an increase in viral replication. This suggests that CD56^dim^perforin^bright^ NK cells are activated and may be capable of killing DENV-infected cells. NKp30 and DNAM-1 potentially play a role in mediating this activation. Although the frequencies of the individual NK cell subsets are unchanged during adult DENV infection, the frequency of CD56^dim^perforin^bright^ NK cells increases in DENV-infected children, suggesting a shift toward a degranulation response. Degranulation is an effective mechanism for killing virally infected cells. However, it is possible that, in children, this response is not specific enough, contributing to pathogenesis rather than viral clearance.

A previous study has shown a significant increase in the percentage of NKG2C^+^ NK cells during acute DENV-2 infection (22). However, we did not observe an increase in the frequencies of the NKG2C-expressing NK cell subsets, CD56^dim^CD57^+^ and CD56^dim^Fas-L^bright^ NK cells, in either DENV-infected children or adults. This could be due to the fact that our analysis was performed on individual NK cell subsets rather than total NK cells. It may also be due to our DENV cohort having a lower percentage of human CMV–positive patients compared with that of Petitdemange et al. (22), which had a seroprevalence rate of 91.7%. Unfortunately, the human CMV status of the patents in our cohort was unavailable.

Using adult cohorts, Azeredo et al. (20) and Gandini et al. (49) have shown an association between NK cell activation and mild dengue. Conversely, Green et al. (19) observed a higher frequency of CD69^+^ NK cells in children who developed dengue hemorrhagic fever compared with children with mild disease. Although our cohort does not contain any patients that could be classified as having dengue hemorrhagic fever, our data supports this idea of NK cells responding differently to DENV infection in children versus adults. We found that expression of NK cell activation and functional markers NKG2D, Syk, Fas-L, perforin, and CD69 was higher in adult dengue patients compared with healthy controls and that expression of all these makers was predictive of adult DENV infection. Although CD69 expression was increased in DENV-infected children and predictive of infection, the NK cell phenotype was also characterized by CD57 expression. Importantly, NKG2A expression was higher in healthy children and significantly predictive of that state. CD57 expression is induced upon NK cell stimulation and increases with age whereas NKG2A expression decreases with age (17, 50, 51). CD57 also defines a subset of NK cells that have a greater cytotoxic capacity and higher sensitivity to CD16 signaling (52). These data suggest that DENV infection induces NK cell maturation in children. In all likelihood, the adults in our cohort have greater immune experience than the children. Consequently, their NK cells are already mature and can mount a more rapid, functional response that may limit viral spread more effectively.

Importantly, our unsupervised clustering analysis complements the differential state tests we performed using a generalized linear model. Occasionally the results from these different analyses appear contradictory. For example, perforin expression was upregulated during adult DENV infection, however there was no corresponding increase in the frequency of the CD56^dim^perforin^bright^ NK cell subset. Similarly, expression of CD57 was upregulated during pediatric DENV infection, however no increase in the frequency of the CD56^dim^CD57^+^ NK cell subset was observed. These results can be explained by the fact that perforin and CD57 expression is likely upregulated across multiple NK cell subsets during acute DENV infection. Consequently, an increase in their expression can be predictive of infection without changing the distribution of the NK cell subsets.

Adult DENV infection led to increased expression of Fas across all myeloid cell subsets. This, along with the fact that adult NK cells upregulate Fas-L, suggests that adult NK cells may kill DENV-infected myeloid cells via the Fas-Fas-L pathway. CD14^+^CD16^−^ and CD141^+^ myeloid cells in DENV-infected children upregulated DR4/DR5 alone or in combination with Fas. Unfortunately, our NK cell CyTOF panel did not include the DR4/DR5 ligand, TRAIL, and additional PBMC samples are not available. Therefore, we cannot determine whether this pathway is contributing to myeloid cell apoptosis in children. A previous study has shown an increase in the percentage of TRAIL^+^ NK cells as well as an increase in TRAIL expression during adult DENV infection (49), making it likely that TRAIL-mediated apoptosis is occurring.

Strikingly, acute DENV infection in adults led to fewer phenotypic changes in myeloid cells than in children. Of the markers whose expression increased during adult DENV infection, many would facilitate NK cell targeting. Besides Fas, two of the myeloid subsets expressed MICA/MICB and/or Nectin-2. MICA and MICB are ligands for the activating NK cell receptor NKG2D. NKG2D’s expression increased during adult DENV infection and was predictive of that state. Nectin-2 is a ligand for the activating receptor DNAM-1, which was expressed on the CD56^dim^perforin^bright^ NK cell subset. Many ligands for activating NK cell receptors were similarly upregulated during pediatric DENV infection. These included DR4/DR5, Fas, Nectin-2, as well as DNAM-1 ligand CD155 and NKG2D ligands ULBP-1, -2, -5, and -6. However, unlike what was observed in the adults, there was also significant upregulation of LLT-1, a ligand for the inhibitory receptor CD161, and HLA class I molecules, which are known to suppress the anti-DENV NK cell response (27–29). Together, these findings suggest that during pediatric DENV infection, myeloid cells adopt a more NK cell–suppressive phenotype than adult myeloid cells. This potentially facilitates DENV-infected myeloid cell evasion of the NK cell response in children.

With the hope of being able to compare the expression of NK cell ligands by infected and uninfected cells, two Abs against DENV proteins were included in our PBMC panel, one against the NS3 protein and another against the E protein. Unfortunately, we were unable to detect DENV-infected cells. In published work and in our experience, both the anti-NS3 and anti-E protein Abs successfully detect DENV-infected cells in the setting of in vitro DENV infection (29, 53). However, the low frequency of infected cells during natural infection could explain this result. Indeed, Zanini et al. (54) were only able to detect viral RNA in the PBMCs of two out of six confirmed dengue patients by virus-inclusive single-cell RNA sequencing. Importantly, viral RNA was only identified in patients who progressed to severe dengue. Based on these results, our inability to detect DENV proteins is unsurprising, especially given that our cohort was entirely made up of mild cases.

This study has limitations. The first is the lack of functional assessments, which did not allow us to directly evaluate whether adult NK cells are more responsive to DENV-infected cells than pediatric NK cells. Regrettably, we do not have sufficient samples to perform such studies. Another limitation is the small sample size of DENV-infected patients, particularly in our NK cell analysis. To analyze the largest sample size possible, we included one adult DENV-1 patient in our analysis. Considering that different DENV serotypes have been reported to differentially modulate expression of certain proteins (55), we were concerned that this might introduce additional heterogeneity into our data. Therefore, we performed our analysis with and without the adult DENV-1 patient. We did not observe differences in the conclusions. This gave us confidence that although the numbers may be modest, this data provides a solid foundation on which to design future studies comparing the adult and pediatric NK cell response to DENV infection.

Overall, our results show that pediatric and adult NK cells are uniquely impacted by acute DENV infection. We discovered that the frequency of total NK cells decreases in DENV-infected children, but not in DENV-infected adults. This decrease in pediatric NK cell frequency is accompanied by an increase in the abundance of a CD56^dim^perforin^bright^ NK cell subset. During adult DENV infection, NK cells develop an activated phenotype, whereas the phenotype of pediatric NK cells is largely one of increased maturation. Similarly, adult myeloid cells upregulate ligands for activating NK cell receptors that may facilitate killing of DENV-infected cells by CD56^dim^perforin^bright^ NK cells and the Fas-Fas-L pathway. In contrast, myeloid cells from pediatric patients upregulate inhibitory ligands, which may suppress the NK cell response. These findings need to be confirmed with a larger DENV cohort that includes longitudinal samples. Additional markers of NK cell function such as IFN-γ and TNF-α should also be evaluated. This will be critical to determining how the observed differences in the anti-DENV immune response in children and adults change across the different phases of infection. It would also be important to determine whether shifts in expression of specific markers or the frequencies of certain immune cell subsets correlate with disease severity. Such analyses may point to age-specific predictors of progression to severe DENV. Finally, functional studies comparing the ability of NK cells derived from children and adults to respond to DENV-infected cells are necessary to determine whether functional differences exist. Overall, this study provides insight into the differences between the NK cell response to pediatric and adult DENV infection, which may have a bearing on disease severity.

## Supplementary Material

1

## Figures and Tables

**TABLE I.** Panamanian DENV cohort demographics

Characteristic	Adult		Pediatric	
	DENV^+^ (*n* = 9)	Healthy (*n* = 31)	DENV^+^ (*n* = 19)	Healthy (*n* = 22)
Age, y, median (range)	29 (21–57)	31 (16–57)	9 (2–13)	7 (2–15)
Females	3	19	8	8
Males	6	12	11	14
Days of symptoms, median (range)	3 (1–5)	N/A	3 (1–5)	N/A

N/A, not applicable.

## Abbreviations used in this article:

CyTOF: mass cytometry
DENV: dengue virus
ICGES: Gorgas Memorial Institute of Health Studies
MSI: mean signal intensity
UMAP: Uniform Manifold Approximation and Projection
