# Supplementary material for: Mass Cytometry Analysis of the NK Cell Receptor–Ligand Repertoire Reveals Unique Differences between Dengue-Infected Children and Adults
Source: Immunohorizons. Author manuscript; Available in PMC 2021 Nov 22. (PMC8608029; doi:10.4049/immunohorizons.2000074)
Supplement: 1 [file NIHMS1756657-supplement-1.pdf]

Specificity	Clone	Isotope
HLA-DR	L243	^{89}Y
CD45	HI30	^{102}Pd
CD45	HI30	^{104}Pd
CD45	HI30	^{106}Pd
CD45	HI30	^{108}Pd
CD19	SJ25-C1	Qdot® 655 (^{112}Cd-^{114}Cd)
CD3	UCHT1	^{115}In
CD20	2H7	^{141}Pr
CD163	GH1/61	^{142}Nd
Pan HLA class I	W6/32	^{143}Nd
CD7	CD7-6B7	^{144}Nd
CD8	SK1	^{145}Nd
CD48	BJ40	^{146}Nd
BDCA-2 (CD303)	201A	^{147}Sm
ICAM-1	HA58	^{148}Nd
LLT-1	402659	^{149}Sm
Flavi E protein	D1-4G2-4-15	^{150}Nd
CD4	OKT4	^{151}Eu
CD64	10.1	^{152}Sm
HLA-B/C	DT9	^{153}Eu
CCR2	K036C2	^{154}Sm
HLA-E	3D12	^{155}Gd
Fas (CD95)	DX2	^{156}Gd
Nectin-1	R1.302	^{157}Gd
MICA/B	159227/236511	^{158}Gd
DR4/5	DJR1/DJR2-2	^{159}Tb
CD1c	L161	^{160}Gd
ULBP-1,2,5,6	170818/165903	^{161}Dy
CD11c	Bu15	^{162}Dy
NS3	E1D8	^{163}Dy
Nectin-2	TX31	^{164}Dy
CD155	SKII.4	^{165}Ho
HLA-Bw4	REA274	^{166}Er
CD32	IV.3	^{167}Er
HLA-Bw6	REA143	^{168}Er
CD14	M5E2	^{169}Tm
CD11b	ICRF44	^{170}Er
LFA-3	TS2/9	^{171}Yb
CD33	WM53	^{172}Yb
CD141 (BDCA-3)	1A4	^{173}Yb
CD56	NCAM16.2	^{174}Yb
CD86	IT2.2	^{175}Lu
B7-H6	875001	^{176}Yb
DNA-1/DNA-2	NA	^{191}Ir/^{193}Ir
Cisplatin	NA	^{194}Pt/^{195}Pt
CD16	3G8	^{209}Bi

Supplemental Table I: PBMC CyTOF panel.

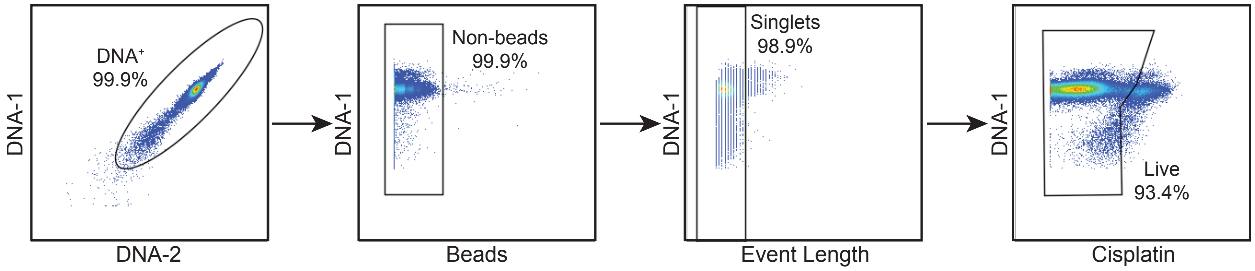

Supplemental Figure 1: Live PBMC gating scheme.

Specificity	Clone	Isotope
CD57	HCD57	^{89}Y
HLA-DR	Tu36	Qdot® 655 (^{112}Cd-^{114}Cd)
CD3	UCHT	^{115}In
CD38	HIT2	^{141}Pr
CD69	FN50	^{142}Nd
CD33	WM53	^{143}Nd
CD14	M5E5	^{143}Nd
CD2	RPA-2.10	^{144}Nd
CD19	HIB19	^{146}Nd
CD8	SK1	^{147}Sm
FcRγ	Polyclonal	^{148}Nd
CD4	SK3	^{149}Sm
Syk	4D10.2	^{150}Nd
CD62L	DREG-56	^{151}Eu
Ki-67	Ki-67	^{152}Sm
KIR2DS4	179315	^{153}Eu
KIR2DS2	Polyclonal	^{154}Sm
NKp46	9E2	^{155}Gd
NKG2D	1D11	^{156}Gd
TIGIT	741182	^{157}Gd
2B4	C1.7	^{158}Gd
DNAM-1	DX11	^{159}Tb
FAS-L	NOK-1	^{160}Gd
NKp30	P30-15	^{161}Dy
Siglec-7	S7.7	^{162}Dy
NKG2C	134522	^{163}Dy
NKp44	P44-8	^{164}Dy
TACTILE (CD96)	NK92.39	^{165}Ho
KIR2DL1	143211	^{166}Er
CD94	DX22	^{167}Er
CXCR6	K041E5	^{168}Er
PD-1	EH12.2H7	^{169}Tm
KIR2DL5	UP-R1	^{170}Er
NKG2A	131411	^{171}Yb
NTB-A	NT-7	^{172}Yb
KIR3DL1	DX-9	^{173}Yb
CD56	NCAM16.2	^{174}Yb
KIR2DL3	180701	^{175}Lu
Perforin	B-D48	^{176}Yb
CD16	3G8	^{209}Bi

Supplemental Table II: NK cell CyTOF panel.

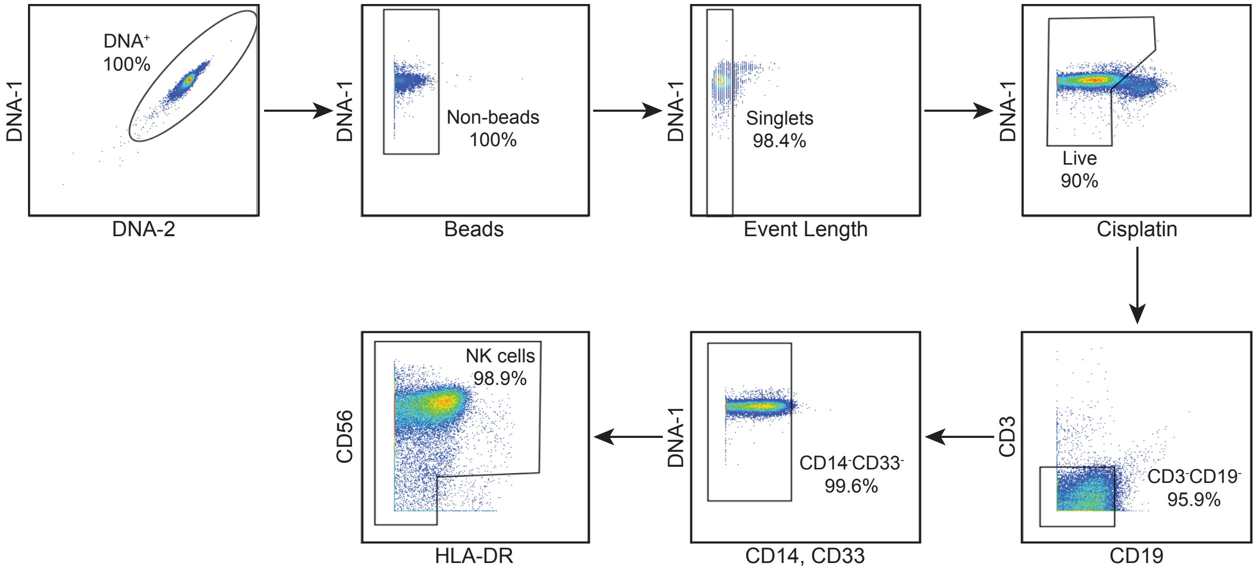

Supplemental Figure 2: NK cell gating scheme.
